# Concomitant Brain Injury and Spinal Cord Injury Management Strategies: A Narrative Review

**DOI:** 10.3390/jpm12071108

**Published:** 2022-07-06

**Authors:** Adriana D. Valbuena Valecillos, David R. Gater, Gemayaret Alvarez

**Affiliations:** 1 Department of Physical Medicine and Rehabilitation, Miller School of Medicine, University of Miami, Miami, FL 33136, USA; dgater@miami.edu (D.R.G.J.); g.alvarez3@med.miami.edu (G.A.); 2 Christine E. Lynn Rehabilitation Center for the Miami Project to Cure Paralysis, Miami, FL 33136, USA; 3 The Miami Project to Cure Paralysis, Miller School of Medicine, University of Miami, Miami, FL 33136, USA

**Keywords:** traumatic brain injury, spinal cord injury, tetraplegia, paraplegia, dual diagnosis

## Abstract

Spinal cord injury (SCI) is a catastrophic event with multiple comorbidities including spastic paralysis, sensory loss, autonomic dysfunction with sympathetic blunting, neurogenic orthostatic hypotension, neurogenic restrictive and obstructive lung disease, neuropathic pain, spasticity, neurogenic bladder, neurogenic bowel, immobilization hypercalcemia, osteopenia/osteoporosis, neurogenic obesity, and metabolic dysfunction. Cervical and thoracic SCI is all too often accompanied by traumatic brain injury (TBI), which carries its own set of comorbidities including headaches, seizures, paroxysmal sympathetic hyperactivity, aphasia, dysphagia, cognitive dysfunction, memory loss, agitation/anxiety, spasticity, bladder and bowel incontinence, and heterotopic ossification. This manuscript will review the etiology and epidemiology of dual diagnoses, assessment of both entities, and discuss some of the most common comorbidities and management strategies to optimize functional recovery.

## 1. Introduction

The World Health Organization (WHO) has recognized neurotrauma as a “critical public health problem” that leads to significant losses to the injured individual, the individual’s family, and the entire community as a result of lifelong disability or death [1]. Traumatic neurologic conditions such as spinal cord injury (SCI) and traumatic brain injury (TBI) are events that globally affect society [2,3]. Challenges to the initial evaluation of patients presenting with altered mental status due to medications, intoxication, and intubation, the immediate focus on apparent SCI or dependency on advanced imaging may delay the dual diagnosis.

The National Spinal Cord Injury Statistical Center estimates the incidence of new traumatic SCIs (TSCI) to be roughly 40 cases per one million, with about 12,000 new cases per year and a prevalence of approximately 273,000, within a range of 238,000 to 332,000 [4]. Similarly, the Global Burden of Disease (GBD) study estimated a TBI incidence of 1.11 million and prevalence of 2.35 million in the United States in 2016 [3]. The Centers for Disease Control and Prevention (CDC) estimates that in 2014, there were approximately 2.53 million emergency department (ED) visits, 288,000 hospitalizations, and 56,800 deaths related to TBI in the United States [5].

The rate of dual diagnosis has been increasing in the USA, accounting for 2.46 per 100,000 admissions in 2008 [6]. Estimates of concurrent TBI in patients with primary traumatic SCI range from 12.5 to 74.2%, based on the diagnostic criteria utilized [7,8,9,10,11]. Severe TBI was the most common presentation, occurring in 73.9% of patients with dual diagnosis in Turkey [11], while mild TBI was the most common presentation evaluated with dual diagnosis in the USA [12]. In China, 7% of comatose patients were found to have concomitant cervical SCI [13]; similarly, in the USA, 8% of patients diagnosed with severe TBI had associated cervical SCI [14]. TBI and SCI are primarily caused by falls and motor vehicle collisions (MVC). It is expected that the incidence of TBI and SCI will surge due to the increase in population density, aging, and the use of motor vehicles, motorcycles, and bicycles, which is concerning because of the specialized care that people with SCI can require [2,3]. The majority of studies showed a high male-to-female ratio and an age of peak incidence younger than 30 years old. Traffic accidents were typically the most common cause of SCI, followed by falls in the elderly population [15]. Similar to isolated SCI and TBI, dual diagnosis more frequently occurred in men and following MVC [6]. A difference in the isolated TBI population was that MVC is more common in young adults, but falls were the most common cause overall [16,17,18]. Alcohol intake has been identified as an important risk factor for traumatic injuries including the SCI, TBI, and dual diagnosis patients [6]. Depending on the risk factors, the incidence of a dual diagnosis may approach 60%, according to the SCI Model Systems [19,20]. The criteria for identifying TBI in most of these studies include post-traumatic amnesia, initial abnormal Glasgow Coma Scale (GCS), and abnormal brain imaging [21]. Similarly, patients will have downstream issues with behavioral problems, neuropsychological impairment, and psychopathology [19,20,22]. While issues such as headaches and seizures are a hallmark of TBI patients, autonomic dysfunction, neurogenic bladder, neurogenic bowel, spasticity, heterotopic ossification, anxiety, and depression can be present in either injury process, and will definitely be a prevalent concern in dual diagnosis patients. The associated issues stemming from a dual diagnosis of SCI and TBI together present a unique challenge to the practicing physiatrist, regardless of their sub-field of expertise [9,20,21,23,24].

## 2. Assessment

The International Standards for the Neurological Classification of SCI (ISNCSCI) should be used to establish the level and completeness of SCI [25]. The initial autonomic assessment should include the International Standards to determine remaining Autonomic Function after Spinal Cord Injury (ISAFSCI) [26], used in concert with the ISNCSCI. This tool documents cardiovascular, bronchopulmonary, and sudomotor symptomatology as well as genitourinary, bowel, and sexual function, providing a clinical “score” of autonomic dysfunction [26].

Detailed initial assessment for brain injury should include confirmation of loss of consciousness (LOC), Glasgow Coma Score (GCS) [27], duration of Post-Traumatic Amnesia (PTA) [28,29], orientation [28,29,30], behavioral issues (Rancho Los Amigos Cognitive Scale) [31], and initial imaging should be conducted to properly assess the patient with dual diagnosis [21,23]. Associated factors that may mask or mimic TBI include intensive care unit (ICU) delirium or psychosis, infections, pain, and psychoactive medications [32]. Confounding clinical neurological indices of TBI such as hypoxia, intoxication (alcohol/other), pre- existing cognitive deficits (e.g., dementia or prior TBI) and intubation, all of which occur with some frequency with SCI, can also alter the LOC and GCS scores [21]. Negative brain imaging on initial imaging can frequently mask the presence of mild or even moderate TBI, and positive findings, especially contusions, may be missed in up to 67% of cases [20,23]. Similarly, the presence of moderate or severe cognitive deficits in patients with TBI could mask SCI due to the lack of accurate evaluation, especially when the initial spine imaging is not confirmatory.

## 3. Management

There are no current guidelines regarding the admission process for dual diagnosis patients. Historically, patients would be assigned to either an SCI or TBI unit based on which diagnosis contributes most to the patient’s functional impairment and barriers to recovery [22]. Patients with dual diagnosis are more likely to manifest behavioral issues, exhibit psychopathology, and have more severe neuro-psychological impairment than patients with SCI alone [19]. Concomitant TBI may delay one’s ability to tolerate 3 h of therapy per day and demonstrate the potential to benefit from rehabilitation interventions, thereby delaying rehabilitation admission. Comorbidities associated with TBI or SCI alone may be masked or exaggerated in dual diagnosis including seizures, headaches, autonomic dysfunction, neurogenic bladder, neurogenic bowel, spasticity, heterotopic ossification, and anxiety/depression.

### 3.1. Seizures

Early post-traumatic seizures are defined as occurring ≤7 days after TBI, and during this period, seizure prophylaxis is recommended; if no seizure has occurred during this period, ongoing risk of post-traumatic seizures is low [33,34]. Late post-traumatic seizure risks associated with brain injury include skull fractures with the penetration of bone or metal fragments, bilateral parietal intracranial hemorrhage (ICH), and midline shift [35]. Of note, 52% of individuals with severe TBI demonstrated seizure activity on continuous EEG monitoring in the ICU setting, without the outward appearance of seizures [36]; concurrent SCI might further confound the issue due to motor paralysis below the level of SCI. Seizures should not occur as the result of SCI; but may present if gamma aminobutyric acid (GABA) agonist medications such as diazepam or baclofen, used for spasticity management, are suddenly withdrawn [37].

### 3.2. Headaches

Post-traumatic headaches are commonly associated with mild (47–95%) and moderate (20–38%) traumatic brain injuries [38], but may vary in the location of pain, with the most frequent location identified as temporal (82%), followed by forehead (76.5%), neck (76%), back of head (53%), eyes (47%), and vertex (29%) [39]. It is important to rule out possible central causes of post-traumatic headache including pneumocephalus, cerebral spinal fluid (CSF) fistulas/leaks, hygromas, or subdural hematoma with appropriate imaging studies. While persons with SCI might also experience muscle tension headaches, they are also likely to experience headaches with autonomic dysreflexia [40] and sleep apnea [41].

### 3.3. Autonomic Dysfunction

Both TBI and SCI can experience autonomic dysfunction, but symptoms may be significantly different based on the location of injury. (Table 1) Autonomic hyperactivity associated with TBI was first described in the medical literature in the 1950s, and since then has been provided with multiple pseudonyms, but in 2007 [42], Alejandro Rabinstein introduced the term “paroxysmal sympathetic hyperactivity”, which gained favor and subsequently consensus in 2014 [43]. Paroxysmal sympathetic hyperactivity (PSH) is characterized by the simultaneity of clinical features that are paroxysmal in nature, demonstrate sympathetic overreactivity to normally non-painful stimuli, persist ≥3 consecutive days, persist ≥2 weeks post-injury, persist despite the treatment of alternative differential diagnoses, the lack of alternative explanations, and respond to medication with a decrease in sympathetic features. Clinical features typically include elevated heart rate, respiratory rate, systolic blood pressure, temperature, sweating, and posturing during the episodes [43]. PSH likely involves a 2-stage process with disconnection of descending inhibitory pathways from supraspinal structures (including the paraventricular nucleus, dorsomedial nucleus, and lateral hypothalamus as well as the rostral ventrolateral medulla), with subsequent resolution as inhibitory drivers are recovered [42,44,45,46]. Treatment goals included finding and avoiding the triggers, mitigating the sympathetic outflow, and addressing the effects of PSH on other organ systems through supportive care. As the majority of triggers involve pain, urinary retention, or movement, avoidance and analgesia are recommended including judicious administration of opioid agonists, GABAA agonists, and gabapentin [44,45,46,47]. Sympathetic outflow may be reduced by α-2 adrenergic agonists, non-selective β-blockers, and certain neuromodulators including bromocriptine for reducing temperature and sweating, baclofen for spasticity and spasms, and dantrolene to treat malignant hyperthermia [44,45,46,47].

Persons with SCI above T6 are likely to experience autonomic dysreflexia (AD) in which a noxious stimuli below the level of injury causes a massive reflex sympathetic outflow, splanchnic vasoconstriction, and hypertensive crisis, often accompanied by reflex bradycardia [40]. Symptoms include a pounding headache, flushing, and sweating above the level of SCI. Treatment includes sitting the person upright, loosening any tight clothing, and removing the noxious stimuli that are most likely associated with bladder or bowel dysfunction, but may include skin lesions, musculoskeletal disorders, or genital causes [40]. By definition, AD involves SBP >20 mm Hg above the baseline, but can increase above 300 mm Hg within minutes, depending upon the stimuli and the person’s unique physiology; pharmacological intervention is often warranted to mitigate the hypertension [40]. Of note, individuals with high thoracic and cervical SCI will often have baseline neurogenic orthostatic hypotension at the baseline and require compression garments, abdominal binders, and medications in order to compensate [26,48]. Persons with higher levels of SCI will also be at risk for hyperhidrosis and thermoregulatory dysfunction due to the sympathetic blunting of the thoracolumbar cord.

Dual diagnoses with autonomic dysfunction can be very challenging, and the consultative services of both TBI and SCI may be required to optimally manage the patient. Of note, PSH is usually self-limiting and resolves within weeks or months of the injury, whereas AD often does not appear during the initial stages of “spinal shock”, in which both musculoskeletal and sympathetic reflexes are dampened; emerging from spinal shock may take weeks or months [26].

### 3.4. Neurogenic Bladder

Very few studies have looked at the difference in the presentation and management of neurogenic bladder in patients with dual diagnosis. A review article compared the most common presentation based on urodynamic analysis. It showed that TBI neurogenic bladder dysfunction was more common in patients with frontal lobe injuries, the coordinated relaxation of the distal sphincter during detrusor contraction was preserved, and the involuntary detrusor contraction (IDC) was the most common urodynamic abnormality thought to be related to the loss of cortical inhibition caused by suprapontine lesions [49]. Additionally, patients that sustained moderate and severe TBI had abnormal urodynamic studies without reporting urinary symptoms, which could be secondary to the associated cognitive deficits [49].

After SCI, the location of the lesion can be described as suprasacral, mixed, or sacral infrasacral [50]. In a suprasacral SCI, the sacral reflex arc and the pontine micturition center (PMC) remain intact, but the SCI prevents communication between them, essentially disinhibiting the sacral micturition reflex arc due to the upper motor neuron (UMN) lesion. The detrusor becomes hyperreflexic, as does the external urethral sphincter, leading to detrusor sphincter dyssynergia (DSD) and very high pressures within the detrusor, putting upper urinary tracts at risk [50,51]. In a mixed SCI such as conus medullaris syndrome involving both cord and cauda equina, supraspinal disinhibition of the sacral micturition reflex may occur, or may be abolished due to damage to the sacral roots required for the micturition reflex; both conditions can lead to urinary incontinence [50,52]. In a sacral/infrasacral SCI, the lower motor neurons are damaged, and the voiding reflex arc is interrupted while the PMC remains intact, leading to an areflexic detrusor muscle and flaccid external urethral sphincter. The presentation and management of neurogenic bladder vary based on the level of SCI; however, it is important to note that even patients with similar levels of SCI may not present in the same way. For these reasons, the American Urological Association (AUA) and Society of Urodynamics, Female Pelvic Medicine & Urogenital Reconstruction (SUFU) Guidelines on Adult Neurogenic Lower Urinary Tract Dysfunction (NLUTD) for Diagnosis and Evaluation [53] as well as treatment and follow-up [54] have recently been provided. History and physical examination, urinalysis, post-void residual (PVR), bladder diaries, non-invasive tests, and invasive tests are all important in the initial evaluation of NLUTD for risk and stratification. Renal and bladder ultrasound can be used to determine a PVR volume, and more critically, to evaluate for upper tract dysfunction. An ultrasound that demonstrates hydroureter or hydronephrosis without underlying upper tract disorders indicates that the storage pressures of the bladder are unacceptably high. This can be a result of either prolonged incomplete bladder emptying, where large, undrained bladder volumes cause excess pressure, or there can be an underlying pressure disorder of the bladder. Namely, this involves either high compliance (Volume/Pressure) of the bladder, or low compliance (Volume/Pressure), as seen in DSD. Untreated, this increased pressure can lead to upper urinary tract damage including hydronephrosis, pyelonephritis, and renal failure [55,56]. Therefore, early recognition of these processes is essential, and renal ultrasound is an important screening tool [53,57,58]. Of note, while persons at moderate-risk for NLUTD only need to have renal imaging once every 1–2 years, annual upper tract imaging is recommended for those at high risk [53]. Urodynamic studies allow for the assessment of lower tract function including the evaluation of detrusor compliance and voiding pressures [50,53,59]. This study involved the placement of a urethral catheter as well as an abdominal catheter (placed either in the rectum or the vagina) that allows for the interpretation of detrusor pressures during filling and voiding. This can be combined with fluoroscopic images to provide anatomic details such as bladder diverticuli, vesicoureteral reflux, bladder outlet obstruction, and stones [50,53,59]. Urodynamic testing is critically important to diagnose conditions that predispose to upper tract damage such as poor bladder compliance, DSD, and bladder outlet obstruction. In suprasacral SCI, where often the underlying pathology is a loss of inhibition of the micturition reflex, this will appear in urodynamics as uninhibited detrusor contractions, whereas in a sacral/infrasacral injury, the detrusor muscle will most likely be either underactive or atonic [60]. Of note, clinicians must hemodynamically monitor individuals at risk for AD throughout the urodynamic study, and the study should be terminated and the bladder drained, if AD occurs. If hemodynamic improvement does not occur with this maneuver, pharmacotherapy should be considered [53]. The bladder volume and pressure at which AD occurred should be documented and considered when providing treatment recommendations. For patients with chronic SCI who are at moderate or high risk NLUTD and experience new signs, symptoms or complications (e.g., recurrent urinary tract infections (UTIs), stones, AD), multichannel urodynamics may be performed to determine the etiology and new treatment strategy [53]. Clean intermittent catheterization (CIC) is regarded as the best practice for preventing UTIs compared to other methods since it does not involve indwelling long-term foreign body use and allows for more natural bladder filling and voiding cycles [54,61], however, if the person has concomitant TBI, cognition and irritability may require temporary use of an indwelling catheter. Pharmacological intervention may be required to reduce detrusor overactivity including the use of antimuscarinics, β-3 adrenergic agonists, or chemodenervation with the botulinum toxin [54]. If bladder outlet obstruction is demonstrated in urodynamics, an α-1 adrenergic antagonist may be required with the close monitoring of hypotension as it is a common side effect of this class and a common factor in both TBI and SCI [54].

### 3.5. Neurogenic Bowel

Gastrointestinal (GI) dysfunction is attributed to higher morbidity and mortality after TBI and SCI, which, however, is often overlooked. In both types of neurotrauma, TBI and SCI, the primary parasympathetic control to the GI tract, the vagus nerve, remains anatomically intact. However, individuals with TBI or SCI are highly susceptible to GI dysfunctions, evidence that suggests that the parasympathetic nervous system (PNS) and the enteric nervous system (ENS) within the gut no longer cooperatively coordinate GI functions, suggesting a disruption in the vago-vagal reflexes [62]. Of the post-TBI GI complications, the most common comorbidity is gastritis with a reported incidence of 74–100 percent [62,63,64], while 68% of individuals with TBI suffer from chronic malnourishment [62,65]. GI dysfunctions contribute to post-TBI morbidity and mortality, although no exact mechanisms have been identified [66]. The neural and autonomic dysregulation is variable across studies, but often results in decreased GI motility among other GI complaints [67]. Sympathetic nervous system (SNS), PNS, and somatic innervation to the external anal sphincter, puborectalis, and pelvic floor muscular to the lower GI tract is similar to that of the bladder/sphincter, but SNS vascular innervation to most of the small and large bowel is mediated through the greater splanchnic nerve that arises from the T7–T8 spinal cord segments [58]. Gut reflexes normally assist with voluntary defecation. When the nervous system is intact, these reflexes may be voluntarily suppressed by supraspinal inhibition and continence is maintained through voluntary contraction of the external anal sphincter, puborectalis, and pelvic floor musculature. Similar to the suprasacral SCI in the bladder, the hyperreflexic GI tract will result in rectosphincter dyssynergia, with constipation and intermittent fecal incontinence, whereas the sacral/subsacral bowel will result in an areflexic bowel, constipation, and fecal incontinence due to flaccid sphincter and pelvic floor musculature. Management strategies for the former include daily or every other day reflex evacuation using chemical (suppository) and mechanical (digital) stimulation, whereas the latter must rely on daily disimpaction. Goals of bowel care include controlled predictable and thorough evacuation, absence of constipation, diarrhea and AD, and bowel continence. Persons with the dual diagnosis of TBI and SCI may have difficulty in understanding the actual bowel care program process and may need cueing and/or assistance to gain confidence in continence.

### 3.6. Spasticity

Both TBI and suprasacral SCI can result in spasticity (i.e., a velocity dependent increase in muscle tone due to exaggerated stretch reflexes) [68]. The muscle spindle sends signals of stretch along Ia afferents to the dorsal horn of the spinal cord that can activate the agonist alpha motor neuron to fire if a sufficient number of excitatory post-synaptic potentials (EPSPs) exceed that of inhibitory post-synaptic potentials (IPSPs) at the axon hillock; commensurate gamma motor neurons to the intrafusal fibers at both ends of the muscle spindle ensure continued sensitivity of the spindle itself [69]. Under normal conditions, supraspinal inhibition from corticospinal, corticoreticular, and dorsal reticulospinal tracts dampens the muscle reflexes through increasing IPSPs along the length of the cord. After TBI or SCI, there is disinhibition of these inhibitory influences as those tracts are damaged, so that below the level of injury, EPSPs abound, causing hyperreflexia and spasticity [69]. Those with severe and/or hypoxic brain injury may have more diffuse and severe spasticity with greater extensor patterns, whereas those with SCI tend to have more flexor spasms [69]. Early intervention is important for both to reduce the risk of painful muscle contractures and pressure injuries. Assessment for spasticity typically includes the history and severity of spasms with the Penn Spasm Score [70], relative resistance to velocity-dependent passive stretch using the Modified Ashworth Score [71], and functional assessment of voluntary movement that may be facilitated or impaired by spasticity. If spasticity is helpful, there is no need to treat, but if it is painful, interferes with mobility, or impairs activities of daily living, treatment is likely required. Modalities, bracing, and stretch including proprioceptive neuromuscular facilitation using agonist/antagonist muscle spindle and Golgi tendon organ physiology to provide reciprocal inhibition of muscles across joints to optimize stretch should be trialed [72]. If unsuccessful, medications including gamma amino butyric acid (GABA)B agonists, α-2 adrenergic agonists, or excitation-contraction inhibitor may be trialed separately or in combination [73]. Baclofen, a GABAB agonist, and diazepam, a GABAA agonist, both work by increasing IPSPs at the anterior horns below the level of injury [74,75]; their side effects include somnolence, which may not serve the person with TBI well. Clonidine is an α-2 adrenergic agonist that decreases EPSPs at the anterior horn, but its potent antihypertensive effect may cause hypotension, which is not well-tolerated by the person with SCI [73,75]. Tizanidine is a more selective α-2 adrenergic agonist that also decreases EPSPs at the anterior horn, but with significantly less hypotension than clonidine [75]. Dantrolene works peripherally on the muscle fibers, binding to the ryanodine receptors, and reducing the calcium release from the sarcoplasmic reticulum, uncoupling the excitation–contraction mechanism. Unfortunately, it also weakens muscles under voluntary control, which may be helpful for the person with TBI who has diffuse spasticity, but can be very limiting for the person with SCI [75]. If oral pharmacology is still insufficient or suffering side effects such as sedation, hypotension, aa programmable intrathecal baclofen (ITB) pump can be surgically implanted at the abdomen with a catheter placed in the intrathecal space for the delivery of highly concentrated baclofen into the cerebrospinal fluid [76,77,78]. The advantages of ITB are that it has a reservoir that can contain up to 3 months of baclofen, and relatively small doses are required as it is directly bathing the spinal cord with medication. Disadvantages include the small risks of surgical implantation and the possibility of mechanical failure, leading to either overdose or withdrawal symptoms; troubleshooting strategies should be discussed in advance and standardized algorithms applied if necessary [78]. If focal spasticity is problematic and involving only a few muscles, neurolysis using phenol to pure motor nerves [73] or botulinum toxin to specific muscles can be especially helpful, but must often be repeated every 3–6 months for ongoing effectiveness [79,80].

### 3.7. Heterotopic Ossification

Neurogenic heterotopic ossification (NHO) is the formation of extra-osseous lamellar bone in soft tissue surrounding peripheral joints below the level of injury, and can occur in the first 1–3 months of TBI or SCI, with an equal incidence of ~10–20% [81,82,83,84]. The clinical presentation usually includes a red, warm, painful (when perceived) and swollen joint with decreased range of motion (ROM), and often precedes laboratory or imaging diagnostic abilities by several weeks, although 3-phase bone scintigraphy can precede X-ray findings by 3–4 weeks [85,86]. Alkaline phosphatase and creatinine phosphokinase are usually the first lab markers to appear (within 2–3 weeks after NHO initiation), but are non-specific markers; similarly, C-reactive protein (CRP), erythrocyte sedimentation rate (ESR), and prostaglandin E2 have been shown to be elevated in NHO. The mechanisms are still somewhat unclear, but likely involve the spasticity-induced migration of mesenchymal bone cells to affected joints; immobilization hypercalcemia may further exacerbate progression [81,83,84,87]. Joint incidence is somewhat similar, with SCI more likely to develop hip > knee > shoulder > elbow NHO, while TBI is associated with hip > shoulder > elbow > knee NHO; 84 these site predilections may be the result of emerging spasticity at these joints. While 3-phase bone scintigraphy remains the “gold-standard” for diagnosis, musculoskeletal ultrasound is gaining favor, as it is cost-effective, readily available at the bedside, and does not expose the patient to ionizing radiation [84]. The NHO treatment standard for many years was etidronate, which is no longer available in the USA, so oral indomethacin 25 mg three times daily is the “go-to” standard, although bisphosphonates are of potential benefit [83,84]. Passive ROM exercises may be beneficial or may contribute to more active NHO; judicious management is recommended [83,84].

### 3.8. Anxiety and Depression

Anxiety and depression can occur in both TBI and SCI and can be especially difficult to manage in dual diagnosis. Somner and Witkiewcz have suggested excellent strategies for managing the dual diagnosis of moderate to severe TBI and SCI, which are summarized in Table 2 [88]; however, there are no current guidelines to date.

## 4. Rehabilitation Outcomes

There is limited research on the impact of concurrent TBI in patients with SCI on functional outcomes. Dual diagnosis patients have more complex factors adversely affecting the functional improvement measures (FIM). The improvement in scores at discharge relative to admission (FIM gain), and FIM efficiency (change/unit time) are likely to be lower relative to an SCI patient without TBI, and the length of stay (LOS) may be longer due to the barriers to discharge to home [21]. In addition, functional cognition and neuropsychological test performance was negatively impacted in cases of moderate to severe TBI, and patients with dual diagnosis have poorer memory and problem-solving skills than those without TBI [9,89]. In paraplegia, severe TBI has contributed to lower admission and discharge FIM motor scores, a longer rehabilitation length of stay, lower functional comprehension problem solving, memory, and the speed of information processing [89]. Based on current data, persons with paraplegia and severe TBI should be provided with modified therapeutic interventions that include extensive family education in the challenges of the co-occurrence of SCI and severe TBI. Moreover, health care payers should be prepared to extend the duration of hospitalization for persons with SCI and co-occurring severe TBI to achieve functional goals similar to those of their peers without co-occurring TBI. The most important factors to return to work of a sample of 30 patients with dual diagnosis was personal motivation and social support, while those who were employed in positions requiring manual labor prior to injury were least likely to return to work [90].

## 5. Conclusions

The dual diagnosis of TBI and SCI is under-recognized and requires special attention to meet the challenge of properly managing these issues. Comorbidities of the two conditions may overlap, causing significant challenges in diagnostic and management strategies. These patients face additional challenges compared to patients with isolated TBI and SCI, and understanding the differences is essential to promote a positive rehabilitation outcome.

## Figures and Tables

**Table 1:** Autonomic dysreflexia and paroxysmal sympathetic hyperactivity (storming).

Autonomic Dysreflexia (SCI at or above T6)	Paroxysmal Sympathetic Hyperactivity
Etiology: SCI at the thoracic level T6 and above.	Etiology: TBI, hypoxic/anoxic injury, subarachnoid hemorrhage, stroke, encephalitis, thalamic lesion, vasculitis, post-partum vasoconstriction, cerebral fat embolism.
Pathophysiology: Results from noxious stimuli, which in turn trigger sympathetic hyperactivity including greater splanchnic nerve, resulting in hypertensive crisis and reflex bradycardia.	Pathophysiology (theory): Absence of central inhibitory pathway mechanism on regulation of afferent information which causes increased stimulation of the sympathetic nervous system.
Symptoms: Sudden increase in blood pressure, pounding headaches, flushing of the skin above the level if the SCI, piloerection, nasal congestion, blurred vision, sweating above the level of SCI.	Symptoms: Tachycardia, elevated blood pressure, respiratory rate, elevated temperature, posturing.
Treatment: Sit patient up, remove noxious stimuli (bladder distention fecal impaction,), treat hypertension (e.g., Nitrates, Hydralazine).	Treatment (Challenging to Manage): Environmental management: e.g., remove Indwelling catheters, suctioning, constipation, remove C collars when possible. Medications (anecdotal): Bromocriptine—Fever dystonia Propranolol:decreases hypertension and heart rate, decrease catecholamines Gabapentin: control autonomic symptoms Intrathecal Baclofen: effective but invasive

**Table 2:** Data adapted from Somner JL, Witkiewicz PM. The therapeutic challenges of dual diagnosis: TBI/SCI. Brain Inj 2004; 18 (12):1297–308.

Rancho 4	Rancho 5	Rancho 6–7
Confused/Agitated	Confused/Non-Agitated, Inappropriate	Confused Appropriate to Automatic Appropriate
Provide Private Room, Restraints only when necessary, Cover brace and feeding tube with shirt/abdominal binder, When possible full attendant care, Minimize overstimulation, Regular nursing care including catheterizations, Maintain sleep wake cycles, Tilt in space wheelchair for pressure relief minimize agitation, Frequent reorientation by staff, Co-treatment with 1 therapist, Patient instruction: short 1 step commands, Therapy on a quiet gym with Rest breaks. Aggressive and inappropriate behavior to be dealt with firmly/instantly, Involve psychology for patient family support and therapy.	Therapy sessions still best in quiet gym, Patient reports well to structure and redirection for task completion, Now able to consistently follow 1–2 step commands, Rest breaks and avoidance of overstimulation. Full time supervision by staff or caregivers still important.	Therapy may now focus more on SCI rehabilitation interventions, Therapy may occur in an open gym, Orientation group, memory logbook, and signs in room are helpful, Checklist and environmental cues useful in planning/initiating necessary SCI activities such as pressure relief and catheterizations, Patient should be able to verbalize why braces and treatments are necessary, Paraparetics should learn to don/doff brace, dress in bed, wheelchair mobility, Family education including patient and family in support group.

## Data Availability

Not applicable.
